# Prognostic Value of PLR in Various Cancers: A Meta-Analysis

**DOI:** 10.1371/journal.pone.0101119

**Published:** 2014-06-26

**Authors:** Xin Zhou, Yiping Du, Zebo Huang, Jun Xu, Tianzhu Qiu, Jian Wang, Tongshan Wang, Wei Zhu, Ping Liu

**Affiliations:** Department of Oncology, First Affiliated Hospital of Nanjing Medical University, Nanjing, China; Baylor College of Medicine, United States of America

## Abstract

**Background:**

Recently, more and more studies investigated the association of inflammation parameters such as the Platelet Lymphocyte Ratio (PLR) and the prognosis of various cancers. However, the prognostic role of PLR in cancer remains controversial.

**Methods:**

We conducted a meta-analysis of published studies to evaluate the prognostic value of PLR in various cancers. In order to investigate the association between PLR and overall survival (OS), the hazard ratio (HR) and its 95% confidence interval (CI) were calculated.

**Results:**

A total of 13964 patients from 26 studies were included in the analysis. The summary results showed that elevated PLR was a negative predictor for OS with HR of 1.60 (95%CI: 1.35–1.90; P_heterogeneity_ <0.001). Subgroup analysis revealed that increased PLR was a negative prognostic marker in patients with gastric cancer (HR = 1.35, 95%CI: 0.80–2.25, P_heterogeneity_ = 0.011), colorectal cancer (HR = 1.65, 95%CI: 1.33–2.05, P_heterogeneity_ = 0.995), hepatocellular carcinoma (HR = 3.07, 95% CI: 2.04–4.62, P_heterogeneity_ = 0.133), ovarian cancer (HR = 1.57, 95%CI: 1.07–2.31, P_heterogeneity_ = 0.641) and non-small cell lung cancer (NSCLC) (HR = 1.85, 95% CI: 1.42–2.41, P_heterogeneity_ = 0.451) except for pancreatic cancer (HR = 1.00, 95%CI: 0.92–1.09, P_heterogeneity_ = 0.388).

**Conclusion:**

The meta-analysis demonstrated that PLR could act as a significant biomarker in the prognosis of various cancers.

## Introduction

For a long time, cancer is one of the leading causes of death and a major public health problem worldwide [1]. In spite of the increased survival rate of cancer patients in the last decades, newer diagnostic methods with improved sensitivity and specificity are necessary for the proper detection and prognosis of cancer [2]. So both clinicians and researchers have made widespread efforts to identify biomarkers that predict progression of the disease, response to treatment, and survival. Nevertheless, currently there are no suitable predictors that can be widely used in clinical settings, and therefore, better predictive biomarkers, especially serum biomarkers for predicting the prognosis of various cancers are urgently needed.

Recently, more and more evidence showed that a systemic inflammatory response could play an important role in the development and progression of cancer [3]–[6]. It is well known that inflammation is closely related to different stages of tumor development, including initiation, promotion, malignant conversion, invasion and metastasis. Furthermore, inflammation also affects immune surveillance and responses to therapy [7]. Peripheral blood tests at the time of diagnosis or before treatment may reflect inflammatory conditions within the tumor. Fortunately, systemic inflammation can be assessed by means of widely available markers such as C-reactive protein (CRP), albumin, Neutrophil Lymphocyte Ratio (NLR) and Platelet Lymphocyte Ratio (PLR) [8]. Among these markers, PLR, a combination of circulating platelet and lymphocyte counts, is a representative index of systemic inflammation. Its prognostic value had been studied in many types of cancers including ovarian cancer [9], colorectal cancer [10] and so on. And now, a series of studies have explored the correlation between PLR and prognosis of various cancers. However, according to their results, the current opinion on the prognostic role of PLR in cancer is still controversial. We therefore conducted this meta-analysis to reveal the prognostic value of PLR in various cancers.

## Materials and Methods

### Search strategy and study selection

A systematic review of the studies about PLR in predicting the prognosis of various cancers was performed. Studies were identified by searching PubMed, Embase and Web of Science databases using the following search terms: PLR, platelet-to-lymphocyte ratio, platelet lymphocyte ratio or platelet-lymphocyte ratio with cancer, neoplasms or tumor and prognosis or outcome. Both free text and MeSH search for keywords were used. The last search was updated in March 12, 2014. The “related information” function was used to broaden the search and all abstracts, full texts and references were reviewed. Study was conducted according to the Preferred Reporting Items for Systematic Reviews and Meta-Analyses (PRISMA) statement [11].

The search was conducted by two authors (Huang and Du). We read titles and abstracts of all candidate articles. Articles that could not be categorized based on title and abstract alone were retrieved for full-text review. Articles were independently read and checked for inclusion criteria of articles in this study. Any disagreements were resolved through consensus with a third investigator (Zhou).

### Inclusion and exclusion criteria

Studies were considered eligible if they met the following criteria: (a) studied patients with any type of cancer; (b) investigated the association of pre-treatment PLR and overall survival (OS); (c) published as a full paper in English. Studies were excluded based on the following criteria: (a) letters, reviews, case reports or laboratory studies; (b) studies had duplicate data or repeat analysis; (c) lack of key information for further analysis; (d) non-human research.

### Data extraction

Two investigators evaluated and extracted the data independently under the guidelines of the Dutch Cochrane Centre proposed by Meta-analysis of Observational Studies in Epidemiology (MOOSE) [12]. For each study, the following information was recorded: first author, year of publication, country of origin, ethnicity, total number of cases, cancer type, stage, treatment strategy, cut-off value, follow ups and HR of PLR for overall survival with its 95% confidence intervals and *P* value.

### Statistical analysis

All the survival results were estimated as the hazard ratio (HR) for each study. If possible, the HR and 95% confidence intervals (95% CI) were obtained directly from each study publication. When the data was not directly reported, a mathematical estimation was done by calculating the necessary data according to the methods published by Parmer et al [13]. Cochran’s Q test and Higgins I-squared statistic were undertaken to evaluate the heterogeneity of pooled results. A p<0.10 for Q-test suggested significant heterogeneity among studies and the random-effects model (DerSimonian-Laird method) was performed to calculate the pooled HRs [14]. Otherwise, the fixed-effects model (Mantel-Haenszel method) was applied [15]. To explore the potential source of heterogeneity among studies, meta-regression was conducted utilizing variables as year of publication, ethnicity, cancer type, analysis method and cutoff value. To validate the credibility of outcomes in this meta-analysis, sensitivity analysis was performed by sequential omission of each individual study using the “metaninf” STATA command. Begg’s funnel plot and the Egger’s linear regression test were conducted to examine publication bias of literatures and a p<0.05 was considered significant. All statistical analyses were performed with STATA software version 12.0 (STATA Corporation, College Station, TX, USA). And all *P* values were two-sided.

## Results

**Figure 1:**
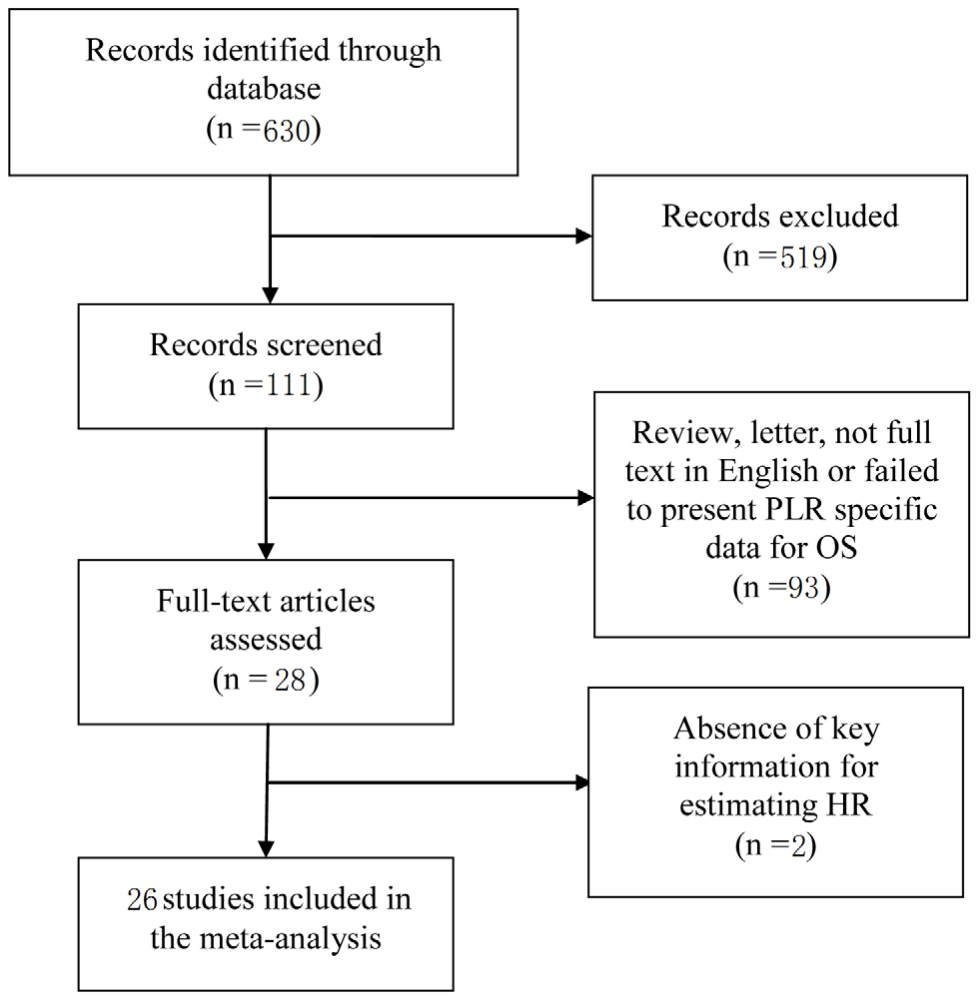
Methodological flow diagram of the meta-analysis.

By the initial search, 630 potentially relevant articles were identified. Then 519 articles were excluded because of obvious lack of relevance. After carefully reading the articles, 95 were excluded (review, letter, non-english studies and studies lack of some data or key information). Finally, 26 articles [9], [10], [16]–[39] were included in this meta-analysis (Figure 1). Authors identified 26 potential studies for full-text review, with excellent agreement between authors. The main features of eligible studies are summarized in Table 1. Among them, participants in ten studies were Asian and in the other twelve were Caucasian. A variety of cancers were recorded in our study, including digestive duct cancer, hepatocellular carcinoma, pancreatic cancer, female reproductive system cancer and non-small cell lung cancer (NSCLC). The cut-off values applied in the studies were not consistent ranging from 100 to 300. Nine studies had a PLR cutoff value of 160 or less, while ten studies used a PLR greater than 160. The remaining seven studies had triple subsets of PLR cutoff, six used 150/300 and one used 100/200. HRs with their 95%CIs were extracted from the graphical survival plots in 4 studies and reported directly in 22 studies, 16 of which calculated HRs by the multivariate analysis and 10 via univariable analysis.

**Table 1:** Main characteristics of eligible studies.

Study	Year	Country	Ethnicity	Number	Cancer	Treatment	Follow-up (month) *	Cut-off
Smith *et al.* [16]	2008	UK	Caucasian	65	Ampullary	Surgery	22.5	160
Aliustaoglu *et al.* [17]	2010	Turkey	Caucasian	168	Gastric	Surgery	NA	160
Bhatti *et al.* [18]	2010	UK	Caucasian	84	Pancreatic	Surgery	NA	100/200
Proctor *et al.* [19]	2011	UK	Caucasian	8759	Mixed	NA	18 (5163)	150/300
Asher *et al.* [9]	2011	UK	Caucasian	235	Ovarian	Surgery	NA	300
Wang *et al.* [20]	2012	China	Asian	324	Gastric	Surgery	39.9 (162)	150/300
Pinato *et al.* [21]	2012	UK	Caucasian	171	MPM	Mix	NA	300
Kwon *et al.* [10]	2012	Korea	Asian	200	Colorectal	Surgery	33.6 (39)	150/300
Wang *et al.* [22]	2012	China	Asian	177	Pancreatic	Surgery	31.33 (132)	150/300
Sánchez-Lara *et al.* [23]	2012	Mexico	Caucasian	119	NSCLC	Chemotherapy	6	150
Carruthers *et al.* [24]	2012	UK	Caucasian	115	Rectal	Preoperative chemoradiation	37.1 (43)	160
Pinato *et al.* [25]	2012	UK	Caucasian	112	HCC	NA	NA	300
Kinoshita *et al.* [25]	2012	Japan	Asian	150	HCC	M	18 (73)	150/300
Raungkaewmanee *et al.* [27]	2012	Thailand	Asian	166	Ovarian	Surgery	14.7 (50)	200
Azab *et al.* [28]	2013	Island	Caucasian	437	Breast	Mix	NA	185
He *et al.* [29]	2013	China	Asian	243	Colorectal	Chemotherapy	21.87 (199)	150/300
Wang *et al.* [30]	2013	China	Asian	111	Cervical	Mix	NA	142.2
Son *et al.* [31]	2013	Korea	Asian	624	Colon	Surgery	NA	300
Fox *et al.* [32]	2013	Australia	Caucasian	362	RCC	Mix	NA	195
Stotz *et al.* [33]	2013	Austria	Caucasian	371	Pancreatic	Mix	NA	150
Lee *et al.* [34]	2013	Korea	Asian	174	Gastric	Chemotherapy	14.9	160
Feng *et al.* [35]	2013	China	Asian	43	SCCE	Surgery	NA	150
Mohamed *et al.* [36]	2013	UK	Caucasian	60	CUP	Mix	NA	300
Unal *et al.* [37]	2013	Turkey	Caucasian	94	NSCLC	Chemoradiation	NA	194
Liu *et al.* [38]	2013	China	Asian	210	NSCLC	Chemotherapy	18.6	152.6
Szkandera *et al.* [39]	2014	Austria	Caucasian	372	Colon	Surgery	68	225

CUP: carcinoma of unknown primary; HCC: hepatocellular carcinoma; Mix: mixed treatment including chemotherapy, hormonal therapy, surgery, radiotherapy, and supportive care; MPM: malignant pleural mesothelioma; NA: not available; NR: upper value not reached; NSCLC: non-small cell lung cancer; RCC: renal cell carcinoma; RFA: radiofrequency ablation; SCCE: small cell carcinoma of the esophagus; TACE: transcatehter arterial chemoembolization.

*The numbers of patients died or lost during the follow up were recorded in parentheses.

**Figure 2:**
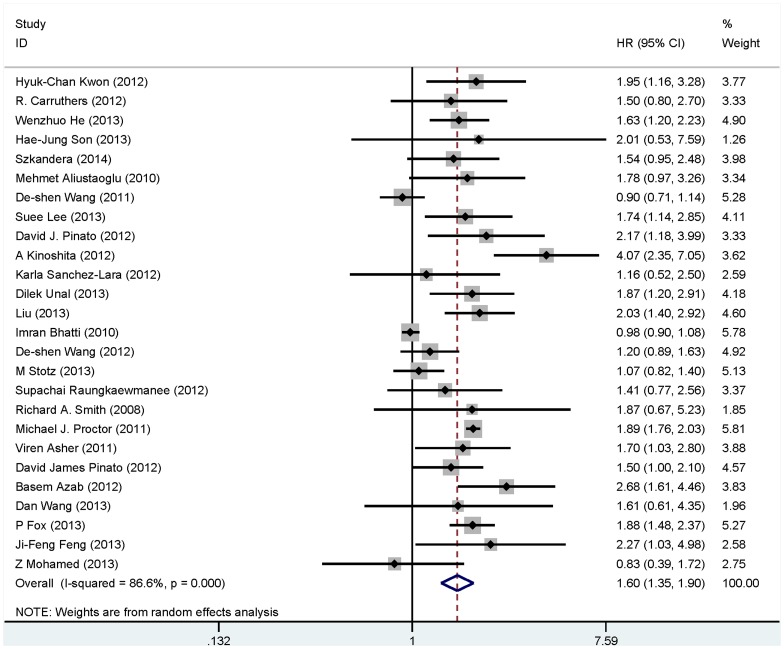
Forrest plots of studies evaluating hazard ratios (HRs) of PLR for overall survival.

The main results of this meta-analysis are listed in Table 2. It is found that elevated PLR predicted a worse outcome for OS with the combined HR of 1.60 (95% CI: 1.35–1.90, P_heterogeneity_ <0.001; Figure 2). Subgroup analyses by ethnicity revealed that negative predictor of PLR for OS was found both in Asian cases (HR = 1.68, 95%CI: 1.28–2.21, P_heterogeneity_ <0.001) and in Caucasian populations (HR = 1.55, 95%CI: 1.24–1.95, P_heterogeneity_ <0.001). When different cancer types were considered, PLR was a negative prognostic marker in patients diagnosed with gastric cancer (HR = 1.35, 95%CI: 0.80–2.25, P_heterogeneity_ = 0.011), colorectal cancer (HR = 1.65, 95%CI: 1.33–2.05, P_heterogeneity_ = 0.995), hepatocellular carcinoma (HR = 3.07, 95% CI: 2.04–4.62, P_heterogeneity_ = 0.133), ovarian cancer (HR = 1.57, 95%CI: 1.07–2.31, P_heterogeneity_ = 0.641) and non-small cell lung cancer (NSCLC) (HR = 1.85, 95% CI: 1.42–2.41, P_heterogeneity_ = 0.451) except for pancreatic cancer (HR = 1.00, 95%CI: 0.92–1.09, P_heterogeneity_ = 0.388). When performing subgroup analyses stratified by analysis method,we found that increased PLR was a negative predictor for OS both by univariable analysis (HR = 1.49, 95%CI: 1.19–1.87, P_heterogeneity_ <0.001) and multivariable analysis (HR = 1.88, 95%CI: 1.59–2.23, P_heterogeneity_ = 0.845). Considering different cutoff values, PLR was a negative prognostic marker for the data applying<or = 160 (HR = 1.55, 95%CI: 1.25–1.92, P_heterogeneity_ = 0.194) and the data applying >160 (HR = 1.76, 95% CI: 1.53–2.02, P_heterogeneity = _0.439). These studies used triple subsets of PLR cutoff revealed the similar results (HR = 1.65, 95%CI: 1.18–2.31, P_heterogeneity_ <0.001).

**Table 2:** Meta-analysis results.

Outcome	Variables	Number of studies	Number of patients	Model	HR (95% CI)	P_heterogeneity_
**OS**	**ALL**	26	13946	Random	1.60 (1.35, 1.90)	<0.001
	**Cancer type**					
	Colorectal	5	1554	Fixed	1.65 (1.33, 2.05)	0.995
	Gastric	3	666	Random	1.35 (0.80, 2.25)	0.011
	HCC	2	262	Fixed	3.07 (2.04, 4.62)	0.133
	NSCLC	3	423	Fixed	1.85 (1.42, 2.41)	0.451
	Pancreatic	3	520	Fixed	1.00 (0.92, 1.09)	0.388
	Ovarian	2	401	Fixed	1.57 (1.07, 2.31)	0.641
	Others	8	10120	Fixed	1.88 (1.76, 2.00)	0.309
	**Ethnicity**					
	Asian	11	2422	Random	1.68 (1.28, 2.21)	<0.001
	Caucasian	15	11524	Random	1.55 (1.24, 1.95)	<0.001
	**Analysis method**					
	Univariable	16	11644	Random	1.49 (1.20, 1.85)	<0.001
	Multivariable	10	2302	Fixed	1.88 (1.59, 2.23)	0.845
	**Cutoff values**					
	< or = 160	9	1376	Fixed	1.47 (1.24, 1.73)	0.194
	>160	10	2633	Fixed	1.76 (1.53, 2.02)	0.439
	150/300	6	9610	Random	1.76 (1.65, 1.88)	<0.001

HCC: hepatocellular carcinoma; OS: overall survival; NSCLC: non-small cell lung cancer.

The results showed that year of publication (p = 0.431), ethnicity (p = 0.782), cancer type (p = 0.208), analysis method (p = 0.200) and cutoff (p = 0.721) did not contribute to the source of heterogeneity.

**Figure 3:**
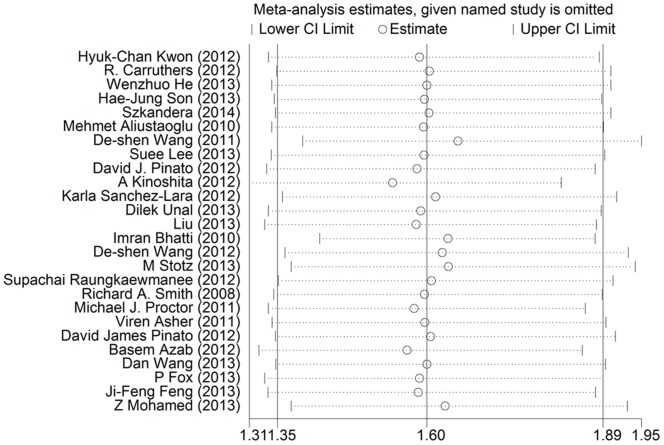
Effect of individual studies on the pooled HR for PLR and OS of patients.

**Figure 4:**
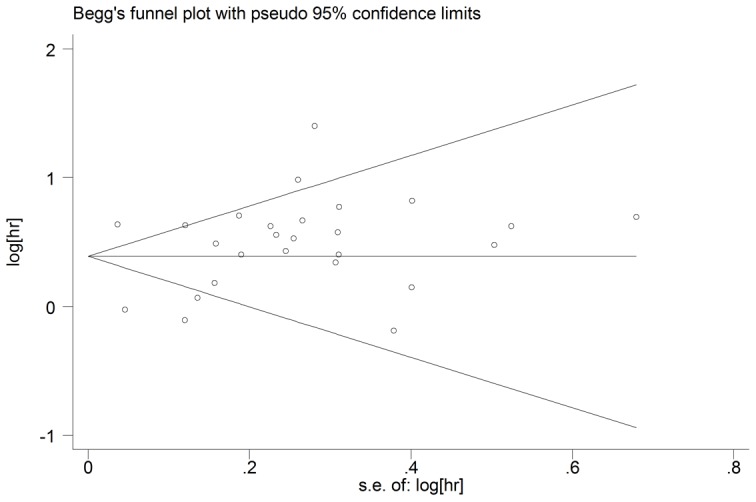
Funnel plots of studies included in the meta-analysis.

We used the leave-one-out sensitivity analyses by removing one study per time to check if individual study influenced the results. The result pattern was not obviously impacted by any single study (Figure 3). Begg’s funnel plot and the Egger’s linear regression test were performed to assess publication bias. The figure of the funnel plot did not show any evidence of obvious asymmetry (p = 0.826; Figure 4). Then, the Egger’s test was performed to statistical test and publication bias was not detected either (p = 0.576).

## Discussion

To date, a variety of predictors have been found and applied in the prognosis of various carcinomas, such as TNM stage, genetic factors, and inflammatory factors. Many inflammatory markers now can be detected in peripheral blood before treatment. Thus, inflammatory marker is a relatively cheap and convenient predictor. Recently, an authoritative article indicated that inflammation with the interaction between various inflammatory cells and extracellular matrix played a crucial role in tumor microenvironment to tumorigenesis [40]. Another study reported that inflammatory cells could release chemicals, notably reactive oxygen species, which were actively mutagenic for nearby cancer cells, accelerating their genetic evolution toward states of heightened malignancy [7]. Additionally, inflammation was evident at the earliest stages of neoplastic progression and was demonstrably capable of fostering the development of incipient neoplasias into full-blown cancers in some cases [41], [42]. Based on these above studies, nowadays many studies investigated prognostic value of various inflammation-based factors including Glasgow Prognostic Score (mGPS) [43], Platelet Lymphocyte Ratio (PLR), Neutrophil Lymphocyte Ratio (NLR) [44], Prognostic Index (PI), and Prognostic Nutritional Index (PNI) [45] in cancer patients. However, the prognostic value of these markers remained inconclusive. Our current study mainly aimed to evaluate the role of PLR in cancer. To our knowledge, it is the first meta-analysis to investigate the prognostic role of PLR in cancers.

The analysis combined the outcomes of 13946 cancer patients from 26 individual studies, indicating that elevated PLR significantly predicted poor OS. Subgroup analyses revealed that worse OS with high PLR could be found both in Asian populations and Caucasian cases. Additionally, elevated PLR was a significant negative prognostic marker for various cancer types. When differently analysis strategies were considered, PLR had prognostic value for poor outcome by univariable analysis or multivariable analysis. Cut off values of PLR used in the enrolled studies were various. As shown in Table 1, a total of 7 studies used triple subsets of PLR and the other 19 studies devided the data into two groups. To evaluate the effect of different cut off values on the prognostic value of PLR, we performed subgroup analyses by cut off values and found that patients with elevated PLR suffered worse overall survival compared to those with low PLR regardless of the different cut off values. The results might strengthen the possibility that PLR could act as a reliable biomarker in predicting clinical outcomes in the future. However, due to the different types and small number of patients, different cut off values obtained from each study might reduce the sensitivity and specificity of the prognostic value of PLR. Thus, future research including more cancer types and more patients to identify widely accepted cut off values for various cancers is warranted. Meta-regression was performed to investigate the source of heterogeneity. However, none of the variables listed above contributed to the heterogeneity in our meta-analysis. In fact, the presence of heterogeneity may result from many factors, including age distribution, gender, tumor size and so on. Much more detailed data was needed to assess the heterogeneity in the future meta-regression.

As shown in Table 2, we can easily learn that PLR is related to prognosis in many cancers, such as colorectal cancer, hepatocellular carcinoma and NSCLC; however, the specific mechanism is still incompletely understood. The relationship of poor prognosis and the elevation of platelets, lymphocytes or their ratio may be explained through an inflammatory process caused by cancer cells. Platelets can promote tumor growth by increasing angiogenesis via the cytokine vascular endothelial growth factor (VEGF) [46]. Wiesner et al. [47] reported that the platelet content of VEGF-A in cancer patients was significantly increased compared to healthy controls. Also some proinflammatory cytokines such as IL-1 and IL-6 promote megakaryocyte proliferation resulting in thrombocytosis [48], [49]. Thrombocytosis has been considered as a negative prognostic marker in several cancers [50], [51]. Meanwhile, platelet aggregation and degranulation along with the consequent release of platelet-derived proangiogenic mediators within the microvasculature of the tumor also could be an important determinant of tumor growth [52]. On the other hand, lymphocytes play a large role in cancer immune-surveillance, which prevent tumor development [53]. Cancer-related inflammation causes suppression of antitumor immunity by recruitment of regulatory T cells and activation of chemokines resulting in tumor growth and metastasis. In breast cancer and melanoma, tumor-infiltrating lymphocytes have been reported as an important prognostic factor, with higher levels associated with better survival [54], [55]. In addition, lymphocytopenia has been reported to be associated with poorer survival outcomes in patients with pancreatic cancer and other gastrointestinal malignancies [56], [57]. The association of clinico-pathological factors and PLR was explored in few studies retrieved in our analysis. Kwon et al. [10] reported that patients with greater PLR showed an increased likelihood of positive lymph node ratio in colorectal cancer. In the study of Asher et al. [9], PLR could reflect residual disease after surgery and status of clinical stage in ovarian cancer which was consistent with the results of Raungkaewmanee et al. [27]. High PLR was also significantly related to bigger size of the tumor and positive status of lymph nodes metastasis in cervical cancer [30]. Azab et al. [28] showed that higher PLR quartiles had significantly higher rates of lymph node involvement, higher rates of metastases, higher AJCC staging and lower hemoglobin in breast cancer patients. Interestingly, Lee [34] found that elevated PLR was frequently observed in female gastric cancer patients who did not accept operation previously and adjuvant chemotherapy. These findings suggest that PLR can be a predictor of the state of some tumors. As mentioned above, thrombocytosis and lymphocytopenia both correlate with the degree of host systemic inflammation that PLR might reflect a novel inflammatory marker incorporating the two hematologic factors [58].

There were several limitations of this study need to be carefully considered. This study was constrained to studies published in English language only. So publication bias cannot be excluded. In addition, heterogeneity among these studies were relatively large that might be caused by different countries, histological type of cancer or/and other factors. In order to reduce the heterogeneity, different cutoff values of PLR or univariate or multivariate regression model have been conducted in our study. Moreover, due to lack of appropriate data, the association of PLR and other important clinical parameters was not explored. Furthermore, most of the patients included in this meta-analysis suffered from digestive system neoplasms. In the future, studies with more types of cancers and larger sample size are needed to present more reliable results.

In conclusion, the meta-analysis firstly shows that an elevated PLR is a negative predictor for survival for various cancers.

## Supporting Information

PRISMA checklist **Checklist S1.**
(DOC)
